# Hepatitis B Flare in Hepatitis B e Antigen-Negative Patients: A Complicated Cascade of Innate and Adaptive Immune Responses

**DOI:** 10.3390/ijms23031552

**Published:** 2022-01-28

**Authors:** Ming-Ling Chang, Yun-Fan Liaw

**Affiliations:** 1 College of Medicine, Chang Gung University, Taoyuan 333323, Taiwan; liveryfl@gmail.com; 2 Division of Hepatology, Department of Hepatogastroenterology, Chang Gung Memorial Hospital, Taoyuan 333423, Taiwan

**Keywords:** HBV, HBeAg, HBV flare, innate immunity, adaptive immunity

## Abstract

Chronic hepatitis B virus (HBV) infection is a dynamic process involving interactions among HBV, hepatocytes, and the host immune system. The natural course of chronic hepatitis B (CHB) is divided into four chronological phases, including the hepatitis B e antigen (HBeAg)-positive and HBeAg-negative phases. During HBV flare, alanine aminotransferase (ALT) levels abruptly rise to >5× the upper limit of normal; this is thought to occur due to the immune response against an upsurge in serum HBV DNA and antigen levels. Hepatitis flares may occur spontaneously, during or after antiviral therapy, or upon immunosuppression or chemotherapy in both HBeAg-positive and HBeAg-negative patients. The clinical spectrum of HBV flares varies from asymptomatic to hepatic decompensation or failure. HBeAg seroconversion with ≥ 1 year of consolidation therapy is accepted as an endpoint of oral antiviral therapy in HBeAg-positive patients, but recommendations for treating HBeAg-negative patients differ. Thus, the management of HBeAg-negative patients has attracted increasing interest. In the current review, we summarize various types of HBV flares and the associated complex cascade of innate and adaptive immune responses, with a focus on HBeAg-negative CHB patients. Hopefully, this review will provide insight into immunopathogenesis to improve the management of HBV flares in HBeAg-negative CHB patients.

## 1. Introduction

Chronic hepatitis B virus (HBV) infection remains a challenging global health problem; approximately 257 million people are chronically infected with HBV [1], and this infection is associated with the risk of hepatic decompensation, cirrhosis, and hepatocellular carcinoma (HCC) [2]. Chronic HBV infection is a dynamic process involving interactions among HBV, hepatocytes, and the host immune system. Based on its virological and clinical manifestations, chronic hepatitis B (CHB) shows a natural course that has been divided into four classical chronological phases: the hepatitis B e antigen (HBeAg)-positive immune tolerance and immune clearance phases and the HBeAg-negative inactive residual and reactivation phases [2,3]. The natural course of chronic HBV infection features intermittent alanine aminotransferase (ALT) elevations and episodic hepatitis flares, which may resolve spontaneously or deteriorate, leading to the development of hepatic decompensation, failure, or even death [4]. Currently, nucleos(t)ide analog (Nuc) is the first-choice therapy for >90% of CHB patients. Accumulating evidence supports the feasibility of finite Nuc therapy in HBeAg-negative patients; thus, the management of HBeAg-negative patients, especially during off-Nuc relapse or hepatitis flares, has attracted increasing clinical and research interest [5,6,7]. However, the underlying immunopathogenesis of HBV flares in HBeAg-negative CHB patients is incompletely understood. In the current review, we describe and summarize various types of HBV flares and their underlying immunological mechanisms and clinical scenarios, with a focus on HBeAg-negative CHB patients. Hopefully, this review will provide insight into immunopathogenesis to improve clinical management and promote the development of new therapeutic approaches for HBV flares in HBeAg-negative CHB patients.

## 2. Overview of Hepatitis Flares in HBeAg-Negative Patients

In early 1980, an HBV flare was defined as “an abrupt ALT elevation > 300 U/L (normal < 40 U/L) in patients with a baseline ALT level < 200 U/L (<5 times the upper limit of normal (ULN))” [8]. Later, this definition was refined to “an abrupt elevation of serum ALT to >5× ULN or a greater than 3-fold increase in ALT, whichever is higher” [9] and then to “intermittent elevations of aminotransferase activity to >10× ULN and more than twice the baseline value” [10]. Notably, a large study showed that the 1-year spontaneous HBeAg seroconversion rate was over 60% in patients with ALT > 5× ULN, in contrast to 5% in those with ALT < 5× ULN [11]. These findings suggest that “an abrupt ALT elevation > 5× ULN” is the minimum criterion of a hepatitis flare, and this ALT level has been widely accepted as a threshold in categorical analyses of clinical studies since the 1990s [3].

### 2.1. Clinical Presentations

**Figure 1:**
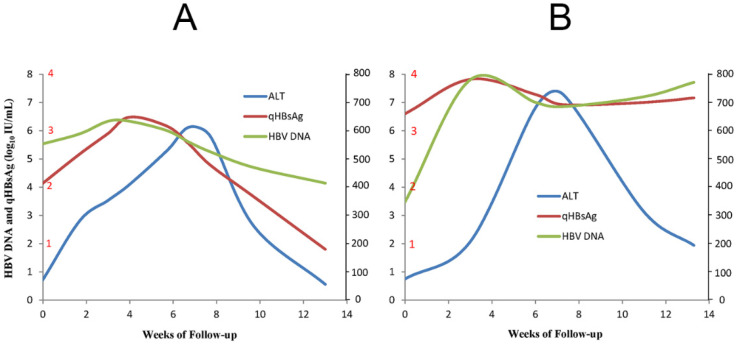
The schematic diagram shows the representative cases of effective immune clearance (**A**) and ineffective immune clearance (**B**). qHBsAg: quantitative HBsAg; ALT: alanine aminotransferase.

Among HBeAg-negative CHB patients, the incidence of flares ranges from 6% to 33% over 2 to 7 years of follow-up [12]. During a typical episode, an upsurge of serum HBV DNA and hepatitis B surface antigen (HBsAg) levels usually precedes the abrupt rise in ALT levels in both HBeAg-positive and HBeAg-negative patients [4]. Within a period of 1 to 2 months, most cases of flare resolve, but some have a more protracted course, and repeated [12,13], sustained (≥ 6 months) [14], or severe (with alpha-fetoprotein (AFP) levels greater than 100 ng/mL and/or bridging hepatic necrosis) flare episodes are more frequently associated with the development of cirrhosis [13]. AFP, a product of specific fetal tissues and neoplastic cells of hepatocyte or germ cell origin in adults, is produced whenever liver cells regenerate [15]. Approximately 25–30% of HBV flare episodes are associated with an increase in serum AFP, the level of which usually peaks 1–2 weeks after ALT levels peak before returning to normal within 3–12 months after the flare [4]. Our previous studies have shown that AFP levels > 100 ng/mL during HBV flares are closely correlated with the presence of bridging hepatic necrosis [16], and an early HBsAg reduction was found to occur in an AFP level- and ALT level-dependent manner in entecavir (ETV)-treated patients, suggesting the impact of hepatic cytolysis rather than Nuc per se [17]. However, as both pregnancy and HCC also lead to elevated AFP levels in HBV-infected patients, any increase in AFP should prompt screening for HCC in HBV-infected patients after the possibility of pregnancy is excluded [4]. Some episodes of flare are followed by HBV suppression, a decline in HBV antigen, and the resolution of hepatitis (Figure 1A). However, other flare episodes involve an increase in HBsAg/HBV DNA levels or persistently high HBsAg/HBV DNA levels despite a decline in ALT (Figure 1B); the first situation is considered to indicate a beneficial or host-dominating flare, and the second indicates a detrimental or virus-dominating flare [3,5,12]. Although hepatitis flares can occur spontaneously, they may occur during or, more frequently, after antiviral therapy in the setting of immunosuppression or immune reconstitution [3]. Below, various types of HBeAg-negative hepatitis flares are highlighted.

#### 2.1.1. Spontaneous Hepatitis Flare

Spontaneous hepatitis flares occur more commonly in HBeAg-positive patients than in HBeAg-negative patients, with a calculated annual incidence of 28.6% and 10.3%, respectively, during an average follow-up of 23.5 months. Spontaneous reactivation of hepatitis B is the major cause of this type of flare in both HBeAg-positive (91.5%) and HBeAg-negative patients (62.5%), and the clinical, laboratory, and histologic findings of these flares were found to be similar in HBeAg-positive and HBeAg-negative patients [18]. Specifically, among asymptomatic HBeAg-negative patients, the spontaneous flare rate varies from 1.46% [19] to 4.3% [15] annually. Precore mutants, male sex, and age ≥ 30 years at presentation are independent predictors of HBeAg-negative flares [20].

#### 2.1.2. Hepatitis Flare during Antiviral Therapy

Hepatitis flares may occur in 2% [12] to 14.9% [21] of HBeAg-negative patients during Nuc therapy; over 90% of hepatitis flare episodes occur within 3 months, and these flares are rarely seen after 1 year. These flare episodes are self-limiting and show a decline in HBV DNA levels during continuing treatment [13,16]. In pivotal studies of peginterferon (pegIFN) alfa 2a for CHB, an ALT > 10× ULN was observed during treatment in 12% of HBeAg-negative patients, although the incidence of HBV flare (i.e., ALT > 5× ULN) was uncertain [22].

#### 2.1.3. Hepatitis Flare after Cessation of Antiviral Therapy

Potent Nucs, such as ETV, tenofovir disoproxil fumarate (TDF), and tenofovir alafenamide (TAF), can suppress HBV DNA to undetectable levels. However, these agents have no direct effect on HBV covalently closed circular DNA (cccDNA), and HBV flares are quite common in HBeAg-negative patients after the withdrawal of Nuc therapy, especially when consolidation therapy is not optimal [5]. Using the “The Asian Pacific Association for the Study of the Liver stopping rule” or more stringent criteria, the reported 1-year incidence of hepatitis flare among HBeAg-negative patients treated with ETV and TDF was 21.7% and 42.4%, respectively [5]. Notably, HBV flares after the cessation of lamivudine (LAM), adefovir dipivoxil (ADV), and TDF mostly occur within 6 months, in contrast to the typical presentation > 6 months after the cessation of ETV therapy [5,23]. Off-therapy severe flares (ALT > 1000 IU/L or flare + bilirubin > 3.5 mg/dl or prolonged prothrombin time) are rare, and hepatic decompensation has been reported in only a few patients with cirrhosis [24]. Factors predictive of relapse or flare in HBeAg-negative CHB patients include age (>40 years) [25], duration of consolidation therapy (hazard ratio = 0.991 per month) [26], a serum HBV DNA level ≥ 2000 IU/mL at 1 year after HBeAg seroconversion [27], baseline [26] and end-of-treatment (EOT) [26,28] HBsAg levels, baseline [28] and EOT [29] hepatitis B core-related antigen (HBcrAg) levels, and EOT pregenomic HBV RNA levels [29].

#### 2.1.4. Hepatitis Flare in the Setting of Immunosuppression

Immunosuppressive medications, such as chemotherapy and corticosteroids, inhibit the immune response and accelerate HBV replication [30,31]. In addition to their immunosuppressive effects, corticosteroids may activate glucocorticoid-responsive elements in the HBV genome to further enhance HBV replication and gene expression. These combined effects result in an increase in viremia [30]. Upon discontinuation of immunosuppressive medications, immune competence is restored, and infected hepatocytes are rapidly destroyed. Theoretically, the more potent the immunosuppression is, the greater the level of viral replication and the clinical consequences of sudden withdrawal [31]. Flare following the withdrawal of steroid therapy alone is generally less severe than flare that occurs during/following chemotherapy for cancer [32]. Rituximab (RTX) is a human–mouse chimeric monoclonal antibody that targets the B cell antigen cluster of differentiation (CD). RTX induces B cell depletion and the subsequent impairment of B cell antigen-presenting function, with consequent reductions in specific anti-HBV CD4^+^ T cell activation and proliferation [33]. HBV reactivation after RTX treatment was found to occur in 8% of HBsAg-negative/hepatitis B core antibody (HBcAb)-positive patients with rheumatoid arthritis (RA), of whom 50% experienced hepatitis flares [34]. Among HBsAg-negative/HBcAb-positive patients with CD20^+^ B cell non-Hodgkin lymphoma (CD20 NHL), 8.9% experienced HBV reactivation within a median follow-up of 24 months after RTX-containing treatment [35]. Although successful monitoring of HBsAg-negative/HBcAb-positive lymphoma patients on RTX with close on-demand antiviral therapy has been reported [36] without adverse liver outcomes, the American Association for the Study of Liver Diseases 2018 Hepatitis B Guidance recommends that HBsAg-negative/HBcAb-positive patients on drugs that target B lymphocytes, such as RTX, are given Nuc prophylaxis [37]. Among patients with HBsAg seroreversion (RS, i.e., HBsAg reappearance), 78.1% were reported to have hepatitis flares. More cycles (≥6) and prolonged durations of RTX therapy, hematopoietic stem cell transplantation (HSCT) [38], interleukin-18 (IL-18) rs243908, and IL-4 haplotype rs2243248~rs2243263 were shown to be associated with HBV-RS among HBsAg-negative patients with CD20 NHL undergoing RTX treatment [39]. A retrospective study of HBsAg-negative/HBcAb-positive patients showed that the 3- and 5-year cumulative incidences of HBV-RS after allo-HSCT were 8.7% and 10.5%, respectively, at a median of 16 months after allo-HSCT. HBV flares developed in 19% of HBV-RS cases, but no affected patients experienced hepatic failure or death [40]. An analysis of 70 subjects with advanced B cell cancer (12 HBsAg-positive and 29 HBsAg-negative/HBcAb-positive) who underwent chimeric antigen receptor (CAR)-T cell therapy revealed HBV reactivation in 2 HBsAg-positive patients and in 1 HBsAg-negative/HBcAb-positive patient, but no HBV flares were noted [41]. In addition to immune suppression, other chemotherapy-related factors are suspected to play a role in HBV reactivation, including intracellular signaling activated in response to DNA damage induced by doxorubicin [42]. The risk of HBV reactivation is highest in those undergoing chemotherapy for hematological malignancies [32], so Nucs should be routinely administered pre-emptively to HBsAg-positive patients with a hematological malignancy before chemotherapy [43]. Male sex [44], young age, lymphoma, and high pre-prophylactic HBV DNA levels [44,45] were associated with off-therapy HBV flares in patients with malignancies.

#### 2.1.5. Hepatitis Flare Following Immune Reconstitution

Worldwide, approximately 10% of patients with human immunodeficiency virus (HIV) infection are estimated to be chronically coinfected with HBV [46]. Hepatitis flares occur in 20–25% of patients coinfected with HIV and HBV after highly active antiretroviral therapy (HAART) [47]. Many HAART regimens show potent anti-HBV activity, and the recommendation is that HBV–HIV coinfected persons are treated with HHART containing TDF [48]; now, TDF has been replaced with TAF in this regimen, which allows better uptake by lymphoid tissue [49]. A young age, high baseline HBV DNA levels [50], and elevated ALT levels before the initiation of HAART [51] are risk factors for immune reconstitution inflammatory syndrome (IRIS)-induced hepatitis flares. Interestingly, IRIS-induced hepatitis flare was found to be an independent predictor of subsequent HBsAg loss [50].

Although most mothers with CHB are HBeAg-positive, we want to briefly mention pregnancy-associated flares that obviously result from immunologic alterations [12]. In pregnant patients with CHB who are treated with Nucs, postpartum flares are common after the cessation of Nuc treatment [52]; however, these flare episodes are often mild and resolve spontaneously [53]. In untreated pregnant females with CHB, spontaneous flares are also mild and self-limited in both the prepartum and postpartum periods [54]. Among HBeAg-negative pregnant females, approximately 30% showed postpartum HBV reactivation, and all events were observed during the first semester after delivery. A prepartum HBV DNA level > 10,000 IU/mL was found to predict postpartum HBV reactivation [55].

The predictors of various types of HBeAg-negative flares are summarized in Table 1.

## 3. Altered Immune Responses in Chronic HBV Infection

In general, immune responses are impaired in chronic HBV infection. Defective innate antiviral function, exhausted T cells, and a tolerogenic liver environment may all contribute to a poor clinical response [56].

### 3.1. HBV Is a Stealth Virus That Affects the Innate Immunity of the Host 

**Figure 2:**
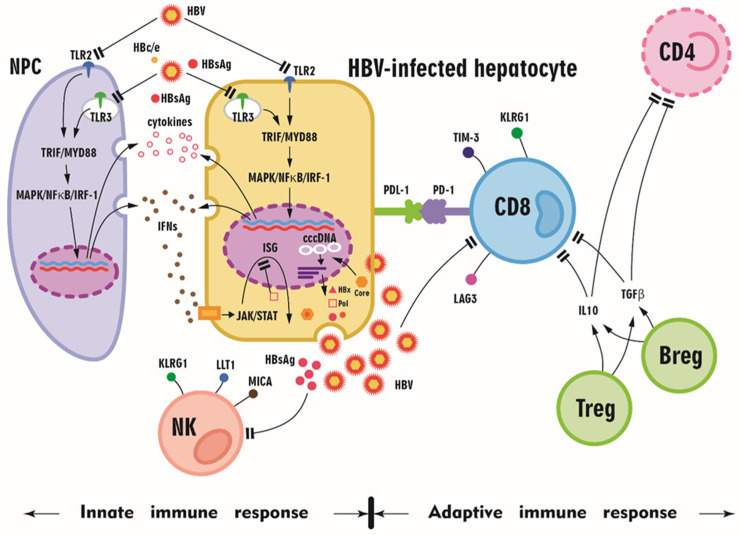
A schematic diagram summarizing the altered innate and adaptive immune responses in HBV-infected patients. Toll-like receptors (TLRs) are expressed in hepatocytes and hepatic nonparenchymal cells (NPCs), including liver sinusoidal endothelial cells, Kupffer cells, and dendritic cells. During viral infection, the stimulation of TLRs by their respective ligands leads to the activation of downstream myeloid differentiation protein 88 (MyD88) and toll/interleukin receptor domain-containing adaptor protein, inducing interferon-β (TRIF)-dependent signaling pathways in hepatic cells and NPCs and the production of proinflammatory cytokines, chemokines, and IFNs. However, HBV and its proteins (HBsAg, HBV core, HBe protein, HBc/e, HBx protein, and HBV polymerase (pol)) might downregulate TLR and downstream signaling, and HbsAg might inhibit natural killer (NK) cells and CD8 cells. The expression of programmed cell death protein 1 (PD-1), T-cell immunoglobulin and mucin domain-containing 3 (Tim-3), killer cell lectin-like receptor G1 (KLRG1), and lymphocyte activating 3 (LAG3) on CD8^+^ T cells illustrates the exhausted status of CD8^+^ T cells upon HBV infection. With interleukin 10 (IL-10) and transforming growth factor beta (TGFβ), regulatory T cells (Tregs) and regulatory B cells (Bregs) inhibit CD8^+^ T cell and CD4^+^ T cell function, and HBV proliferation subsequently accelerates. MAPK: mitogen-activated protein kinase; NF-κB nuclear factor κB; IRF-1: interferon-regulatory factor 1; IFNs: interferons; cccDNA: covalently closed circular DNA; PD-L1: programmed cell death ligand 1; JAK-STAT: Janus kinase-signal transducer and activator of transcription; LLT1: lectin-like transcript 1; MICA: MHC class I polypeptide-related sequence A.

The activation of innate immunity is a prerequisite for proper adaptive immune responses. Toll-like receptors (TLRs) are pattern recognition receptors (PRRs) that function as the first line of antiviral innate immunity because they initiate intracellular signaling pathways to induce antiviral mediators, such as interferons (IFNs) and other cytokines. For example, TLR-mediated signaling pathways are essential for eliciting functional HBV-specific CD8^+^ T cell responses in vivo [57]. Interleukin 12 (IL-12), an essential mediator of liver sinusoidal endothelial cell-mediated CD8^+^ T cell immunity, is produced at a low but sustainable level after TLR2 stimulation [58], and tumor necrosis factor alpha (TNF-α) primarily initiates the innate immune response and triggers adaptive immune responses [59]. Generally, TLR2 and TLR4 signaling results in the activation of intracellular pathways, including the mitogen-activated protein kinase (MAPK) and phosphatidyl-inositol 3-kinase/serine-threonine kinase (PI3K/Akt) pathways, in hepatocytes and reduces HBV replication in an IFN-independent manner [60]. In addition, once an IFN receptor is bound and the signaling pathway is activated, all IFNs induce the recruitment and phosphorylation of Janus-activated kinase (JAK)/signal transducer and activator of transcription (STAT) signaling pathway components, ultimately leading to the nuclear translocation of various proteins to induce downstream gene expression. In particular, IFNs induce the expression of hundreds of IFN-stimulated genes (ISGs) [61]. However, HBV is considered a stealth virus because negligible innate immune responses are induced due to a lack of recognition by PRRs, such as TLRs, or the suppression of IFN production or signaling despite detection by PRRs, although controversy concerning the mechanism of HBV immune escape persists. HBV proteins, such as HBsAg, HBeAg, HBx, and HBV polymerase, are associated with the inhibition of the TLR and retinoic acid-inducible gene I (RIG-I)-like receptor (RLR) signaling pathways, leading to impaired IFN production [62]. Liver specimens from patients with HBV infection did not express more IFN or ISGs than those from control patients, indicating that chronic HBV infection does not activate the innate immune response. Nevertheless, the innate immune response was not suppressed in these CHB tissues, which did produce IFN and induce ISG expression following TLR3 activation upon infection with viruses other than HBV. These findings support the hypothesis that HBV is invisible to PRRs [63]. However, HBV was shown to counteract TLR3 and TLR2/4 activity through the downregulation of TLR expression and attenuation of cellular signaling pathways [60] (Figure 2). Consistently, TLR2 function was reported to be restored in CHB patients who underwent Nuc therapy [64]. Moreover, TLR3 restoration was noted in CHB patients with a pegIFN- or ETV-induced sustained virological response (SVR), although this restoration was more pronounced with pegIFN [65].

Natural killer (NK; CD3^−^CD56^+^) cells are another major component of the cellular infiltrate in the human liver, comprising 30–40% of total intrahepatic lymphocytes, and account for 15% of lymphocytes in peripheral blood [66]. NK cells are important effectors of innate antiviral immunity. In addition to their direct killing of virus-infected cells without antigen-specific priming, they regulate the adaptive immune response by producing IFN gamma (IFN-γ), TNF-α, and immunoregulatory cytokines. HBV replication has differential effects on NK cell ligands, suggesting potential escape mechanisms, such as the upregulation of the inhibitory NK cell ligand LLT1 and the downregulation of the NK group 2D ligand MICA [67]. Furthermore, NK cell activation was shown to be negatively correlated with patient HBsAg levels, NK cell function was correlated with patient age, and NK cell cytotoxicity was correlated with ALT levels [68]. Moreover, reshaping of the NK cell pool toward more CD56-bright cells was also observed. NK cells in patients with low HBsAg levels (<100 IU/mL) simultaneously showed an activated phenotype with increased expression of the activation markers CD38 and granzyme B and the proliferation marker Ki-67 but defective functional responses (macrophage inflammatory protein-1β and CD107a) [68].

### 3.2. Exhausted Adaptive Immune Responses in CHB Patients

CD8^+^ T cells are widely recognized as the main players in viral clearance in acute hepatitis B (AHB) due to the production of the proinflammatory cytokines IFN-γ and TNF-α, which initiate and modulate immune responses through perforin, granzymes, and the Fas lytic pathway to kill virus-infected cells [31,69]. In contrast, CHB patients usually exhibit exhausted HBV-specific CD8^+^ T cell responses. This exhaustion phenotype is characterized by poor cytotoxic activity, impaired cytokine production, and high expression of multiple inhibitor molecules, such as programmed cell death-1 (PD-1), lymphocyte activation gene-3 (LAG-3), and cytotoxic T lymphocyte-associated antigen-4 (CTLA-4) [57]. In addition, coinhibitory receptor killer cell lectin-like receptor G1 (KLRG1) expression on NK cells and antigen-experienced T cells has been postulated to be a marker of senescence [70]. Upregulation of the coinhibitory receptor T cell immunoglobulin and mucin domain-containing protein 3 (Tim-3) defines a subgroup of dysfunctional tumor antigen-specific CD8^+^ T cells [71]. Hyperexpression of PD-1, LAG-3, CTLA-4, KLRG1, or Tim-3 is thus a hallmark of exhausted T cells, as these factors have a negative impact on T cell activation and function. Moreover, intrinsic T cell defects induced or compounded by defective antigen presentation in the liver, such as upregulation of the proapoptotic molecule BCl2-interacting mediator (BIM), mediate the deletion of HBV-specific CD8^+^ T cells. The liver nutrient milieu, populations of immunoregulatory cells, and high levels of the immunosuppressive cytokines IL-10 and transforming growth factor beta (TGF-β) can hinder the ability of T cells to expand and survive, thereby attenuating viral control [72]. Hence, BIM-mediated apoptosis may also contribute to CD8^+^ T cell exhaustion and impede their response to persistent viral replication [73] (Figure 2). Notably, although CD8^+^ T cell exhaustion hampers viral control during chronic HBV infection, the pool of CD8^+^ T cells is phenotypically and functionally heterogeneous. For example, C-X-C chemokine receptor (CXCR) type 5 (CXCR5)^+^CD8^+^ T cells, especially the intrahepatic CXCR5^+^ subset, retain effector functions characterized by the enhanced production of HBV-specific IFN-γ and IL-21. This subpopulation also displays more potent antiviral activity than the CXCR5^−^ subset. Moreover, C-X-C motif chemokine ligand 13 (CXCL13) is a critical chemokine for the recruitment of CXCR5^+^CD8^+^ T cells [74].

The major subtypes of CD4^+^ T cells include T-helper (Th) 1 cells, which produce IFN-γ, IL-2, and IL-12 and promote cellular immune reactions; Th2 cells, which mainly produce IL-4, IL-5, and IL-10 and enhance the humoral immune response [75]; Th17 cells, which produce IL-17/IL-21 (dependent upon RORγT); and regulatory T cells (Tregs), which depend on FoxP3. In particular, CD4^+^ T cells have been shown to be potent producers of IL-21. This pleotropic cytokine is expressed mainly on activated human CD4^+^ T cells, such as T follicular helper (Tfh) cells and Th17 cells [76], and regulates the differentiation, proliferation, and activation of T cells, B cells, and NK cells [77]. Moreover, IL-21 production by Tfh cells was shown to facilitate IgG class switching in B cells and the diversification of the HBV-specific T cell response, including increased IFN-γ production [78]. Hence, in HBV-infected patients, virus-specific CD8^+^ T cells appear to lose their ability to produce IL-2, TNF-α, and IFN-γ and display other hallmarks of exhaustion, including high levels of CD43 and PD-1, in the absence of IL-21 production by CD4^+^ T cells [79].

As humoral immune cells, B cells play a critical role in anti-HBV antibody production. Some studies have also characterized other B cell subsets that are involved in antigen presentation and regulate the immune response in a manner beyond antibody secretion. As B cells act as antigen-presenting cells for CD8^+^ T cells, which limit the expansion of HBV infection in hepatocytes, depletion of B lymphocytes by RTX may disrupt CD8^+^ T cell-induced killing of HBV-infected hepatocytes, thereby allowing virus replication [35]; this hypothesis is supported by the observation of HBV reactivation and hepatocellular injury in patients receiving RTX [12]. However, not all B cell subsets promote the immune response to chronic HBV infection; various B cell subsets jointly mediate persistent HBV infection, tolerance, and liver damage [80]. For example, B cells have been identified as potent regulators of T cell immune responses. IL-10 is the primary factor through which B cells modulate other immune cells, and contributory mechanisms include costimulatory molecule (CD80/CD86) engagement, antigen presentation, and stimulation of the Treg population. In contrast, IL-10-producing B cells are protective in the settings of autoimmune disease, chronic intestinal inflammation, and allograft survival. In humans, analogous regulatory B cell (Breg) subsets were recently shown to suppress CD4^+^ T cell proliferation and IFN-λ and TNF-α production by CD4^+^ T cells and to regulate TNF-α release by monocytes. These suppressive effects are mediated by IL-10 and are independent of TGF-β (Figure 2). Moreover, IL-10 inhibition in vitro rescued polyfunctional virus-specific CD8^+^ T cell responses [81]. Another study showed that increased numbers of circulating IL-10^+^ Bregs and CD4^+^CXCR5^+^Foxp3^+^ follicular regulatory T (TFR) cells are associated with poor virus eradication and liver injury in CHB patients and that serum IL-10 levels are associated with hepatitis flare [82].

Myeloid-derived suppressor cells (MDSCs) also mediate immunoregulatory functions through various mechanisms. MDSCs were suggested to play a role in controlling HBV flares in CHB based on the increased frequency of circulating granulocytic (g) MDSCs in CHB patients with reduced ALT levels, an inverse correlation between the gMDSC frequency and the hepatic necro-inflammatory score, and a dynamic rise/fall in the gMDSC frequency preceding HBV flares. In particular, arginase I is expressed by both MDSCs and hepatocytes and can suppress antiviral T cells through L-arginine depletion [83].

A summary of the altered immune responses in chronic HBV infection is shown in Figure 2.

## 4. Immune Responses in HBV Flare

HBV flares result from enhanced immune responses to an HBV upsurge in patients with CHB, but the triggers of spontaneous HBV flares remain unclear. However, in many instances, precipitating factors for HBV flares can be readily identified and should be anticipated as they may confer the need for prompt antiviral therapy [31], such as in off-Nuc flares or immunosuppressed conditions.

### 4.1. Roles of Chemokines in HBV Flares: Mediators Involved in Both Innate and Adaptive Immune Responses

Studies suggest that increases in HBV DNA and HBV antigen expression over a threshold concentration may activate immune responses prior to a hepatitis flare, during which cytokines and chemokines may also increase and exert both pro- and anti-inflammatory effects [12]. In particular, CXC chemokines are potent attractants of neutrophil granulocytes, T cells, and NK cells [84]. Chemokine (C-X-C motif) ligand 9 (CXCL9; a monokine induced by IFN-γ [MIG]), CXCL10 (IFN-γ-inducible protein 10 [IP-10]), and CXCL11 are all selective ligands of CXCR3. These ligands are usually expressed at low levels under homeostatic conditions but are upregulated by IFN-γ, IFN-α/β, or TNF-α. CXCR3 is highly expressed on activated T cells, memory T cells, and NK cells. CXCL9 induction is restricted to IFN-γ, and this chemokine predominantly functions to recruit CD8^+^ cytotoxic T lymphocytes (CTLs) [85]. In contrast, CXCL10 is strongly induced by IFN-γ and IFN-α/β [86] and is responsible for recruiting innate lymphocytes, including NK and NKT cells, Th1 cells, and effector CD8^+^ T cells [87]. Both CXCL9 and CXCL10 are potent chemoattractants of activated T cells [88]. CXCL10 has been shown to exert hepatoprotective activity, such as inhibiting liver damage and promoting liver regeneration [89]. Thus, CXCL10 might play a role in both inducing inflammation and preserving liver viability. Hepatocytes produce CXCL9 or CXCL10 at an early stage of infection to attract dendritic cells (DCs), NK cells, and memory T cells. A temporary increase in serum CXCL9 and CXCL10 levels has been reported in HBV flares, suggesting their proinflammatory roles in this setting, regardless of HBeAg positivity [4,90,91]. CXCL11 is induced by IFN-γ and IFN-β and weakly induced by IFN-α [92]. Both AHB and CHB patients exhibit increased CXCL9, CXCL10, and CXCL11 levels at peak ALT levels [91]. Altogether, CXCL9, CXCL10, and CXCL11 are induced by HBV-positive hepatocytes with coincident NK cells and plasmacytoid DCs (pDCs), which synergistically produce IFN-α and IFN-γ in response to HBV [91].

### 4.2. Roles of T Cells in HBV Flares

#### 4.2.1. CD8^+^ T Cells: Crucial Players upon the Reversal of Exhaustion

AHB patients characteristically mount a strong multispecific CTL response that is effective at eradicating viruses. Although CTL responses in CHB patients are usually undetectable, they do occur in those with hepatitis flares [93]. Specifically, class I human leukocyte antigen (HLA-I)-restricted CD8^+^ T cells are thought to be critical for hepatitis flares in CHB. Strong simultaneous expression of HLA-I and intercellular adhesion molecule I on the hepatocyte membrane occurs at sites of necroinflammation and active cellular infiltration [94,95]. In addition to cytotoxic cytolysis, HBV-specific T cells might inhibit HBV replication without direct cell contact through the secretion of IFN-γ and TNF-α, thereby inducing the additive deamination of cccDNA and interfering with its stability [96]. In particular, apolipoprotein B editing complex 3 (APOBEC3) cytidine deaminases have been shown to hyperedit HBV DNA and inhibit HBV replication. APOBEC3 gene expression is strongly stimulated by both IFN-α and IFN-γ [97]. The reversal of T cell exhaustion by HBV suppression with antiviral therapy illustrates the role of CD8^+^ T cells in HBV flares. For example, the development of HBV flares during the early period of Nuc therapy is related to the enhancement of the antiviral T cell response, which coincides with a rapid and marked reduction in HBV DNA. This T cell response starts at week 1–2 and lasts until week 20–24 of Nuc therapy [12]. Among HBeAg-positive CHB patients treated with telbivudine or LAM, both viremia and HBeAg drive PD-1 expression, resulting in T cell impairment, and Nuc-induced HBV suppression inhibits PD-1 expression [98]. In HBeAg-negative CHB patients who discontinued long-term Nuc therapy, PD-1^+^CD8^+^ T cells were positively correlated with baseline HBsAg levels. In patients who subsequently achieved HBsAg loss, T cells were less exhausted and displayed greater proliferative capacity after treatment discontinuation, as the T cells expressed low levels of KLRG1 and PD-1 and high levels of Ki-67 and CD38. PD-L1 inhibition could further augment these HBV-specific T cell responses [99]. Moreover, the status of HBV-specific CD8^+^ T cell exhaustion varies with different clinical entities. After HBV peptide stimulation, HBV-specific CD8^+^ T cells in active CHB and HBV–HCC patients expressed less IFN-γ, TNF-α, and CD107a and included fewer activated PD-1^−^Tim-3^−^ cells than asymptomatic subjects [100]. On the other hand, T cells specific to the HBV core protein and polymerase, but not the envelope protein, were selectively enriched within the PD-1^+^ T cell population in HBeAg-negative CHB patients without flares, and these T cells were functional in terms of their proliferative capacity and ability to produce IFN-γ. PD-1 expression on HBV-specific T cells might favor survival by limiting excessive cellular activation, and HBV envelope-specific T cells were reported to be deleted during the course of chronic infection due to high antigenic loads [101]. The reversal of T cell exhaustion through inhibition of the PD-1/programmed cell death ligand 1 (PD-L1) axis improves specific anti-HBV T cell responses in human intrahepatic T cells. Consistently, nivolumab, a PD-1 inhibitor, was shown to be well tolerated in virally suppressed HBeAg-negative patients and led to a decline in HBsAg in most patients and a sustained HBsAg loss in 1 of 22 patients [102].

#### 4.2.2. CD4^+^ T Cells: Dominant Th1 Response

The importance of CD4^+^ T cells in HBV flares is highlighted by IRIS in patients coinfected with HBV and HIV, as both HIV and HBV infections reduce CD4^+^ cell counts [103,104,105]; moreover, the rapid onset of hepatitis flares with an increased CD4^+^ T cell count after commencing HAART suggests the possibility of IRIS [106]. Specifically, seroclearance in chronic viral infection is thought to be mediated primarily by IFN-γ release, but the CD4^+^ T cell pool may simultaneously exacerbate immunopathology via the production of TNF-α, which can worsen hepatic injury [107]. TNFα-producing HBV-specific CD4^+^ T cells were found to be dominant in HBeAg-positive patients with a high viral load, and IFNγ-expressing HBV-specific CD4^+^ T cells dominated in patients with HBeAg seroconversion, HBsAg loss, and viral clearance. Notably, a differentiation process involving a switch from TNF-α-producing to IFN-γ-producing HBV-specific CD4^+^ T cells was observed during flares with HBV clearance [108]. Consistent with the ideas that the IL-12-mediated induction of Th1 cytokines is important for viral clearance in CHB [75] and that the Th1 response is critical in HBV flares, such flares can occur during pregnancy but are more common postpartum due to the suppression of Th1 responses and induction of Th2 immunity, accompanied by the reduction in CD8^+^ T cells during pregnancy, which help the fetus escape immune detection [109]. Among untreated pregnant CHB patients, peripartum flares with increased Th1 cytokines were either mild, suggesting an aborted antiviral immune response [110], or severe [111]. Interestingly, asymptomatic HBV carriers with high levels of viral replication are characterized by Th2-type immune responses. IL-12, the dominant cytokine that specifically promotes Th1 cell differentiation and suppresses Th2 function, is produced by phagocytic cells, B cells, and other antigen-presenting cells. IL-12 stimulates IFN-γ production by peripheral blood mononuclear cells and augments NK cell cytotoxicity and the antigen-specific proliferation of CD8^+^ T cells. Consistently, CHB patients were found to have higher IL-12 levels than controls. A longitudinal analysis during IFN-α treatment revealed a further substantial increase in bioactive IL-12 and Th1 cytokines in patients who cleared HBV with HBeAg seroconversion, and the IL-12 peak followed the hepatic cytolysis peak by 9.8 ± 2.8 weeks and occurred before or simultaneously with HBe seroconversion [75]. Moreover, a study of patients with CHB experiencing a spontaneous flare showed a close temporal correlation between IL-10 levels and fluctuations in viral load or liver inflammation [81].

#### 4.2.3. Remaining Issues Regarding HBV-Specific T Cells during an HBV Flare

Of note, there is an ongoing debate as to whether circulating HBV-specific T cells correlate with HBV disease activity. For example, a study assessing peripheral T cell effector and regulatory responses in 200 adults with CHB and 20 uninfected individuals showed that HBV persists, with virus-specific and global T cell dysfunction mediated by multiple regulatory mechanisms, including those involving circulating HBeAg but without distinct T cell-based immune signatures of the clinical phenotypes [112]. With regard to studies that examined HBV flares after Nuc cessation, a study of 15 patients with HBeAg-negative CHB surveyed the T cell responses at the end of Nuc therapy and 4, 8, and 12 weeks thereafter and found that the T cell phenotype of CHB patients on long-term Nuc therapy was markedly different from that of healthy individuals but was only slightly altered after therapy was discontinued. In some but not all CHB patients, HBV replication relapse after Nuc therapy cessation may alter the T cell phenotype and enhance the responsiveness of HBV-specific T cells to in vitro peptide stimulation [99]. Another study of 27 patients with noncirrhotic HBeAg-negative CHB and complete viral suppression over 3 years showed that an increased frequency of functional HBV-specific CD8^+^ T cells at baseline was associated with sustained viral control off treatment but not with HBV flares. These HBV-specific T cell responses persisted but did not increase after treatment withdrawal [113]. Consistently, a study with 2 distinct cohorts of 19 and 27 CHB patients showed that the absence of hepatic flares following the discontinuation of Nuc treatment correlated with the presence of HBV core and polymerase-specific T cells within the ex vivo PD-1^+^ population [101]. These findings suggest additional T cell regulatory mechanisms of CHB immunopathogenesis that warrant further investigation.

### 4.3. Roles of B Cells in HBV Flares

As an important part of the adaptive immune response, B cells have various immune functions, including antibody secretion, antigen presentation, and immune regulation, that mediate chronic HBV infection [80]. In particular, HBsAb might participate in forming immune complexes and bind DCs to induce a T cell response, anti-HBc IgG binds HBcAg to induce hepatocyte lysis via the classic complement activation pathway initiated by C1, and self-antibodies participate in autoimmune responses that aggravate liver inflammation [80]. Of note, a study of 36 CHB patients showed that HBcAg-specific B cells are present at a higher frequency than those specific for HBsAg. Unlike HBsAg-specific B cells, HBcAg-specific B cells efficiently mature into antibody-secreting cells in vitro. HBcAg-specific B cells are preferentially IgG^+^ memory B cells. However, despite their phenotypic and functional differences, HBcAg- and HBsAg-specific B cells from patients with CHB share an mRNA expression profile that differs from that of global memory B cells and is characterized by the high expression of genes indicative of cross-presentation and innate immune activity. These data suggest that HBV-specific B cells have additional roles beyond antibody production [114]. Another study of 118 treatment-naïve and 34 Nuc-treated patients with CHB and 23 healthy HBsAg-vaccinated controls revealed higher HBcAg-directed B cell responses in HBV clinical phases with elevated ALT levels, irrespective of HBeAg status, while HBsAg-directed responses were lower and did not fluctuate. Viral suppression and ALT normalization upon treatment led to a reduction in HBcAg-specific B cell responses, accompanied by a progressive decrease in serum anti-HBc antibodies [115], in contrast to transiently restoring HBV-specific T cells during Nuc-induced viral suppression [116]. Some patients harbor HBcAg-specific B cell clones that can induce acute cell lysis through a T cell-independent antibody/complement-mediated mechanism, namely, antibody-dependent cell-mediated cytotoxicity (ADCC) [117,118,119,120]. Extreme HBc-specific B cell-associated ADCC can lead to acute liver failure, a serious clinical syndrome that leads to death or liver transplantation in 80% of cases [117,118,119,120]. Moreover, B cells play an important role in presenting HBcAg to activate a T cell-mediated immune response, which might contribute to achieving HBV clearance during an HBV flare [121].

We would like to stress that most of the studies of human HBV immunopathogenesis have focused on the peripheral blood compartment. These studies have revealed HBV-specific and global T cell dysfunction mediated by multiple regulatory mechanisms, but distinct T cell-based immune signatures of clinical phenotypes are lacking [112]. In contrast, studies assessing liver samples have shed light on the clinical implications of immune responses. For example, in one study, functional assays that allow direct ex vivo quantification of circulating and liver-infiltrating HBV-specific CD8 cells showed a high frequency of intrahepatic HBV-specific CD8 cells in the absence of hepatic immunopathology while virus-specific T cells accounted for a lower proportion of cells in liver infiltrates from viremic patients but were present at a similar absolute number because of massive cellular infiltration. Inhibition of HBV replication was associated with the presence of a circulating reservoir of expandable CD8 cells. These results indicate that in the presence of an effective HBV-specific CD8 response, the inhibition of virus replication can be independent of liver damage [122]. Specifically, this landmark study showed that ALT flares are associated with bystander infiltration by non-virus-specific T cells in the liver. Another study utilized traditional immunological assays in conjunction with analyses of global non-antigen-specific immune populations in CHB patients to show that the absence of hepatic flares following the cessation of Nuc therapy correlated with the presence of HBV-specific T cells with high PD-1 expression [101]. Moreover, a recent study utilized imaging mass cytometry to detect immune markers in liver biopsies and found that the hepatic densities of most adaptive and innate immune subsets significantly correlated with serum ALT level, histological fibrosis, and inflammation [123]. Altogether, these findings suggest that HBV-specific T cells in CHB patients likely participate in maintaining microenvironmental homeostasis while coexisting with HBV, as these T cells are too exhausted to expel the virus. When homeostasis is disrupted by a high HBV viral load or the reversal of T cell exhaustion, such as upon viral suppression by Nuc therapy, various immune cells, including non-HBV-specific T cells, may be recruited to the liver to elicit an immune response. The close interplay between the hepatic innate and adaptive immune subsets contributes to liver inflammation and results in hepatitis flares.

**Figure 3:**
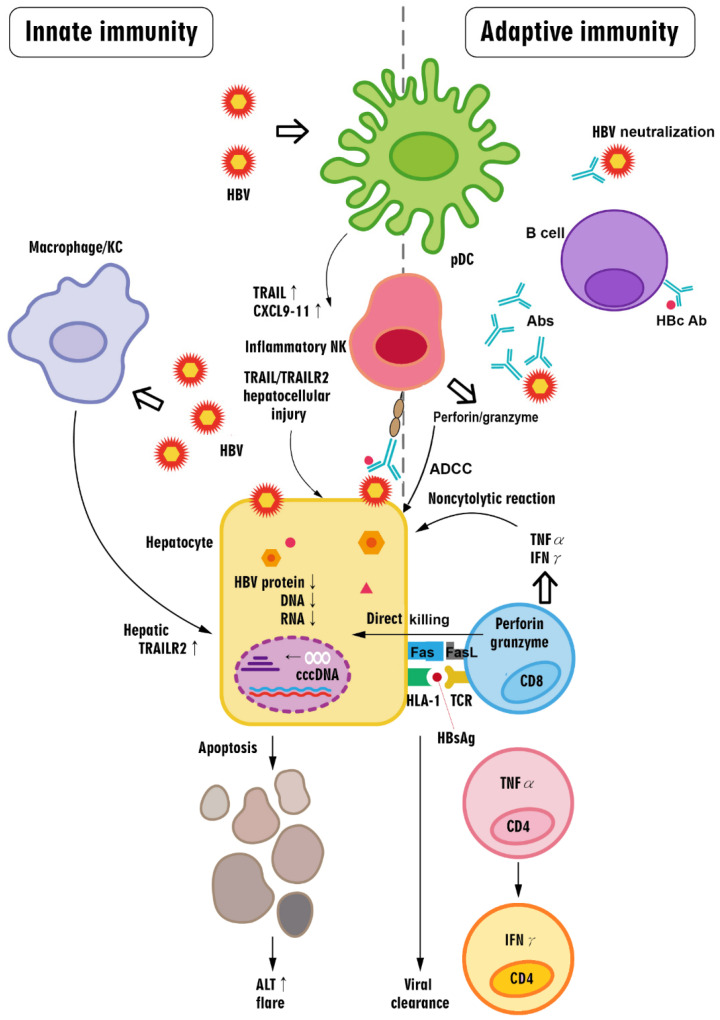
The cascade of innate and adaptive immune responses in HBV flares. Briefly, in HBV flares, innate immune cells in the liver may be activated by the upsurge of HBV or HBsAg above a certain threshold, leading to an increase in cytokines that activate NK cells and promote inflammation while promoting hepatocyte death; these responses can be aggravated by a CD8^+^ T cell-centered immune response. Antibody production by B cells might aid in expelling HBV during an HBV flare via virus neutralization and antibody-dependent cell-mediated cytotoxicity (ADCC), which is mainly executed by hepatitis B core (HBc)-specific B cells. In contrast, HBV-specific CD8^+^ T cells recognize and cause the death of HBV-infected cells via the Fas ligand- and perforin/granzyme-induced pathways. Activated CD8^+^ T cells produce cytokines, including TNF-α and IFN-γ, that evoke the profound pleiotropic downregulation of the virus (including HBV proteins, RNA, and DNA) in a noncytopathic manner in HBV-infected hepatocytes. CD8^+^ T cells also directly kill HBV-infected hepatocytes through perforin and granzyme. Immune-related hepatocyte destruction manifests as ALT elevation (i.e., hepatitis flare). During the process of viral clearance, HBV-specific TNF-α-producing CD4^+^ T cells can differentiate into HBV-specific IFN-γ-producing CD4^+^ T cells. Abs: antibodies; pDCs: plasmacytoid dendritic cells; NK cell: natural killer cell: TRAIL: tumor necrosis factor (TNF)-related apoptosis-inducing ligand; CXCL9-11: CXC motif chemokine ligands 9, 10, and 11; KC: Kupffer cell; TRAILR2: TRAIL receptor 2; FasL: Fas ligand; HLA-1: human leukocyte antigen class I; TCR: T cell receptor; cccDNA: covalently closed circular DNA; HBcAb: hepatitis B core antibody.

The cascade of innate and adaptive immune responses in HBV flares is summarized in Figure 3.

### 4.4. Altered Immune Responses in HBV Flares in HBeAg-Negative Patients

In general, the immunopathogenesis of HBeAg-negative and HBeAg-positive flares, including chemokine alterations and innate and adaptive immune responses, is similar. Given the current interest in hepatitis flares in HBeAg-negative patients, the immunologic features of HbeAg-negative flares are specifically summarized below.

#### 4.4.1. Roles of CXCL8, CXCL9, and CXCL13 in HBeAg-Negative Flares

CXCL8 (IL-8), a chemokine produced by macrophages, epithelial cells, and endothelial cells, can recruit granulocytes, NK cells, and T cells to sites of inflammation [124] and may interfere with the antiviral effect of IFN-α [125]. CXCL8 likely plays a role during AHB and CHB [90]. Consistently, HBeAg-negative patients had higher CXCL8 transcripts in the liver than HBeAg-positive patients, and HBV was shown to activate CXCL8 gene expression by targeting the epigenetic regulation of the CXCL8 promoter; thus, CXCL8 may contribute to reducing HBV sensitivity to IFN-α [126]. Among HBeAg-negative CHB patients with <2.5 × 10^7^ IU/mL HBV DNA, pretreatment with >80 pg/mL CXCL9 was associated with an SVR to pegIFN. In contrast, the SVR rate was low in patients with >2.5 × 10^7^ IU/mL HBV DNA regardless of baseline CXCL9 levels [127]. Moreover, a study of ETV-treated HBeAg-negative patients with a supervised machine learning approach showed that the combination of IL-2, CXCL9, chemokine (C-C motif) ligand 5, stem cell factor, and TNF-related apoptosis-inducing ligand (TRAIL) was reliable for predicting viral relapse [128]. Conversely, after cessation of therapy, baseline serum CXCL9 > 80 pg/mL and on-treatment HBsAg decline were associated with a lower risk of virological relapse in pegIFN-treated HBeAg-negative patients [129], whereas the cytochrome P450 27B1-rs4646536 CT/CC genotypes were associated with more prevalent virological relapse [130]. In a prospective study of 15 HBeAg-negative patients, 13 experienced virological relapse after cessation of Nuc therapy. HBV DNA rebound was associated with increases in plasma TNF, IL-10, IL-12p70, and CXCL10 levels and a subsequent decline in HBsAg [131]. CXCL13 signals through the G protein-coupled chemokine receptor CXCR5 to drive the development of secondary lymphoid tissue and B cell and Tfh trafficking to germinal centers, which leads to the differentiation of B cells into plasma cells and memory B cells [132]. Concomitant increases in CXCL13 and IL-21 were significant in HBeAg-negative CHB patients who attained HBsAg seroconversion with sequential therapy (consisting of a combination of Nucs and subsequent pegIFN-α (48 weeks)) while increases in CXCL13 and IL-21 were not observed in CHB patients who failed to attain HBsAg loss, even during hepatitis flares [133]. Moreover, increased serum CXCL13 and IL-21 levels were reported to correlate with HBsAg loss following an HBV flare after antiviral therapy [64].

#### 4.4.2. Innate Immune Responses

##### Potential Link between the Interleukin 28B Gene (IL28B) and HBeAg-Negative Flares

IFN-α and IFN-β are secreted by almost all virus-infected cells, including hepatocytes, and by specialized blood lymphocytes. In contrast, IFN-γ production is restricted to cells of the immune system, such as NK cells, macrophages, and T cells [59]. Numerous IFN-γ-regulated genes are expressed in the liver during viral clearance, and the upregulation of these genes in the liver results from the adaptive T cell response, as specific T cells that infiltrate the liver are major producers of IFN-γ [60]. Genome-wide association studies (GWASs) have identified single nucleotide polymorphisms (SNPs) at or near the IL28B region on chromosome 19, which encodes IFN lambda 3 (IFN-λ3). IL28B-rs10853728 CC was found to be associated with spontaneous HCV clearance [134]. Likewise, in HBeAg-negative patients, the IL28B-rs10853728 CC genotype was reported to correlate with increased hepatitis activity, suggesting a link between IL28B genotypes and the intrinsic host antiviral immune response to HBV infection [135]. In addition, another GWAS identified a potential association between PRELI domain-containing 2 and G3BP stress granule assembly factor 2 genotypes and the therapeutic response to pegIFN in HBeAg-negative patients [136], although whether these genotypes are associated with HBV flares remains unknown.

##### Cessation of Nuc Treatment Augments NK Responses in HBeAg-Negative Flares

NK cells can regulate specific antiviral immunity by contributing to liver inflammation through TRAIL- and Fas-mediated death and the direct killing of HBV-specific CD8^+^ T cells, which both trigger the recruitment of inflammatory cells that amplify hepatic damage [66,67]. In Nuc-treated HBeAg-negative CHB patients, cessation of Nuc treatment significantly augmented the cytotoxic responses of NK cells. Moreover, this increased NK cell functionality correlated with ALT flares in these patients and was particularly enhanced in patients who presented with HBsAg seroclearance at long-term follow-up [137].

#### 4.4.3. Adaptive Immune Responses

##### High Abundance of HBV Core-Specific CD8^+^ T Cells in HBeAg-Negative Flares

HBV-specific CD8^+^ T cells are crucial for suppressing viral replication, and HBV flares are associated with the expansion and activation of HBV-specific memory cells; notably, more HBV core antigen 18-27-specific CD8^+^ T cells (c18-27-CD8Ts) were detected in HBeAg-negative CHB patients than in HBeAg-positive CHB patients, and hepatitis flares were found to be closely associated with the expansion of c18-27-CD8Ts [138]. Consistently, adoptive transfer of immunity is effective at clearing HBV from HBeAg-negative and serum HBV DNA-negative CHB patients, as evidenced by the HBsAg clearance following hepatitis flares in these patients after they received a bone marrow transplant from HBsAb-positive bone marrow donors [139].

##### Reversal of Adaptive Immune Responses in HBeAg-Negative Patients after Nuc Cessation

Among HBeAg-negative patients who stopped long-term Nuc therapy, the absence of hepatitis flares correlated with the presence of HBV core- and polymerase-specific T cells within the ex vivo PD-1^+^ population [91]. Thus, PD-L1 inhibition further enhanced HBV-specific T cell responses. Consistently, T cells from patients with HBsAg loss expressed low levels of KLRG1 and PD-1 and high levels of Ki-67 and CD38 [99]. Moreover, the presence of functional HBV-specific T cells at baseline was associated with successful sustained viral control after treatment withdrawal [113].

Table 2 summarizes the reported immune responses in studies of HBeAg-negative flares.

## 5. Concluding Remarks

While Nucs are efficient at decreasing the HBV viral load and controlling CHB severity, the issue of addressing hepatitis flares in HBeAg-negative CHB patients remains a major hurdle in the management of CHB. Understanding the immunopathogenesis of flares will pave the way for the development of management strategies. The underlying processes might be revealed by surveying many clinical cases of HBV flares in HbeAg-negative CHB patients during or after Nuc therapy and in those with immunosuppression or immune reconstitution. Regardless of the etiology, all instances of HBV flare initiate from the upsurge in HBV DNA/HbsAg and subsequently elicit an immune response. The exhausted immune cells are rescued by temporal viral suppression through antiviral therapy. After the cessation of antiviral therapy, elements of the innate immune response, including TLRs, NK cells, IFN, and other downstream pathways, and of the adaptive immune response, including CD8^+^ cells, CD4^+^ cells, and B cells, contribute to eliciting HBV flares, reflecting a struggle between HBV and the host. Good or bad of flares signify the host or the virus as the winner in this battle, respectively. In patients with effective immune clearance (Figure 1A) during an HBV flare, the increased HBsAg levels start to decline as ALT increases to its peak and then further decrease with subsequent ALT normalization to achieve remission. Conversely, HBsAg levels increase further with increasing ALT levels or remain high in patients with ineffective immune clearance (Figure 1B), and immediate therapy is indicated to prevent decompensation or even death [3]. Targeting the immunological niches to transform bad instances of flare into good ones in HBeAg-negative CHB patients holds promise to achieve a functional cure for the majority of CHB patients.

## Figures and Tables

**Table 1:** Predictors of various HBeAg-negative HBV flares.

	Spontaneous Hepatitis Flare	Hepatitis Flares after Stopping Antiviral Therapy	Hepatitis Flares in the Setting of Immunosuppression	IRIS-Hepatitis Flares
**Sex**	Male sex [20]		Male sex [44]	
**Age**	> 30 years [20]	> 40 years [25]	Young age [44,45]	Young age [50]
**HBV DNA**	Precore mutants [20]	HBV DNA levels ≥ 2000 IU/mL at 1 year post HBeAg seroconversion [27]	High pre-prophylactic HBV DNA levels [44,45]	High baseline HBV DNA levels [50]
**HBV RNA**		EOT HBV pgRNA levels [29]		
**HBV Ag**		Baseline and EOT HBsAg levels [26,28],		
		Baseline and EOT HBcrAg levels [28,29]		
**Others**		duration of consolidation therapy [26]	Lymphoma [44,45]	ALT levels before the initiation of HAART [51]

IRIS: immune reconstitution inflammatory syndrome; EOT: end of treatment; pgRNA: pregenomic RNA; HBcrAg: hepatitis B core-related antigen; HAART: highly active antiretroviral therapy.

**Table 2:** A summary of the altered innate immune responses in HBeAg-negative flares.

Chemokines	CXCL8	CXCL9	CXCL13
	Upregulation [125]	Upregulation [126,127,128]	Upregulation [64,132]
**Innate immunity**	**IFN**	**NK**	
	IL28B-rs10853728 CC genotype [134]	TRAIL- and Fas-mediated death [66,67]	
		NK cell cytotoxic responses [136]	
**Adaptive immunity**	**T cells**		
	Higher c18-27-CD8Ts [137]		
	PD-L1 inhibition enhanced HBV-specific T cell responses [99]		

CXCL: chemokine (C-X-C motif) ligand; IFN: interferon; NK cell: natural killer cell; c18-27-CD8Ts: HBcAg 18-27-specific CD8 T cells.

## Data Availability

The datasets used and/or analyzed during the current study are available from the corresponding author on reasonable request.

## Abbreviations

HBV: hepatitis B virus; CHB: chronic hepatitis B; HBeAg: hepatitis B e antigen; ALT: alanine aminotransferase; HCC: hepatocellular carcinoma; Nucs: nucleos(t)ide analogs; ULN: upper limit of normal; HBsAg: hepatitis B surface antigen; PegIFN: peginterferon; ETV: entecavir; TDF: tenofovir disoproxil fumarate; TAF: tenofovir alafenamide; cccDNA: covalently closed circular DNA; LAM: lamivudine; ADV: adefovir dipivoxil; EOT: end-of-treatment; HBcrAg: hepatitis B core-related antigen; RTX: rituximab; CD: cluster of differentiation; RA: rheumatoid arthritis; NHL: non-Hodgkin lymphoma; RS: HBsAg seroreversion; HSCT: hematopoietic stem cell transplantation; IL-18: interleukin-18; CAR: chimeric antigen receptor; HIV: human immunodeficiency virus; HAART: highly active antiretroviral therapy; IRIS: immune reconstitution inflammatory syndrome; TLRs: toll-like receptors; PRRs: pattern recognition receptors; IFNs: interferons; TNF-α: tumor necrosis factor alpha; MAPK: mitogen-activated protein kinase; PI3K/Akt: phosphatidyl-inositol 3-kinase/serine-threonine kinase; JAK: Janus kinase; STAT: signal transducer and activator of transcription; ISGs: IFN-stimulated genes; RIG-I: retinoic acid-inducible gene I; RLRs: RIG-I-like receptors; SVR: sustained virological response; NK: Natural Killer; IFN-γ: interferon gamma; PD-1: programmed cell death-1; LAG-3: lymphocyte activation gene-3; CTLA-4: cytotoxic T lymphocyte-associated antigen-4; KLRG1: killer-cell lectin like receptor G1; Tim-3: T cell immunoglobulin and mucin-domain containing-3; BIM: BCl2-interacting mediator; TGF-β: transforming growth factor beta; CXCR5+: C-X-C chemokine receptor type 5+; CXCL13: C-X-C motif chemokine ligand 13; Th: T-helper; Treg: regulatory T; Tfh: T follicular helper; Breg: regulatory B cell; TFR: follicular regulatory T; MDSCs: myeloid-derived suppressor cells; g: granulocytic; CXCL9: C-X-C motif ligand 9; MIG: monokine induced by IFN-γ; IP-10: IFN-γ-inducible protein 10; CXCR3: C-X-C motif chemokine receptor 3; DCs: dendritic cells; pDCs: plasmacytoid dendritic cells; AHB: acute hepatitis B; CTL: cytotoxic T lymphocyte; HLA-I: class I human leukocyte antigen; PD-L1: programmed cell death ligand 1; CXCL8: chemokine (C-X-C motif) ligand 8; TRAIL: TNF-related apoptosis-inducing ligand; IFN-α: Interferon alpha; IFN-β: interferon beta; IFN-γ: interferon gamma; SNPs: singlenucleotide polymorphisms; IL28B: interleukin 28B; IFN-λ3: IFN lamda 3; c18-27-CD8Ts: HBV core Ag 18-27-specific CD8+ T cells.
